# Medical student selection criteria and socio-demographic factors as predictors of ultimately working rurally after graduation

**DOI:** 10.1186/s12909-015-0359-5

**Published:** 2015-04-14

**Authors:** Ian B Puddey, Annette Mercer, Denese E Playford, Geoffrey J Riley

**Affiliations:** 1 Faculty Office, Faculty of Medicine, Dentistry and Health Sciences, University of Western Australia, 35 Stirling Hwy, Crawley, WA 6009 Australia; 2 School of Primary, Aboriginal and Rural Health Care, Faculty of Medicine, Dentistry and Health Sciences, University of Western Australia, 35 Stirling Hwy, Crawley, WA 6009 Australia

**Keywords:** Medical school, Selection criteria, Rural medical workforce

## Abstract

**Background:**

We have previously demonstrated that both coming from a rural background and spending a year-long clinical rotation in our Rural Clinical School (RCS) have independent and additive effects to increase the likelihood of medical students practicing rurally following graduation. The current study assesses the extent to which medical school selection criteria and/or the socio-demographic profile of medical students may further facilitate or hamper the selection of students ultimately destined for the rural medical workforce.

**Methods:**

The study comprised 729 students, admitted from secondary school since 1999 and having graduated by 2011, whose actual workplace location in 2014 was classified as either urban or rural using the Australian Health Practitioner Regulation Agency database. Selection factors on entry (score from a standardised interview, percentile scores for the 3 components of the Undergraduate Medicine and Health Sciences Admission Test (UMAT) and prior academic performance as assessed by the Australian Tertiary Admissions Rank) together with socio-demographic factors (age, gender, decile for the Index of Relative Socioeconomic Advantage and Disadvantage (IRSAD)), were examined in relation to ultimate rural destination of practice.

**Results:**

In logistic regression, those practicing in a rural location in 2014 were more likely to have come from the lower 6 IRSAD deciles (OR 2.75, 95% CI 1.44, 5.23, P = 0.002), to be older (OR 1.86, 95% CI 1.09, 3.18, p = 0.023) and to have a lower UMAT-3 (Non-verbal communication) score (OR 0.98, 95% CI 0.97, 0.99, P = 0.005). After further controlling for either rural background or RCS participation, only age and UMAT-3 remained as independent predictors of current rural practice.

**Conclusions:**

In terms of the socio-demographic profiles of those selected for medical school entry from secondary school, only older age weakly augmented the selection of graduates likely to ultimately work in a rural destination. Among the selection factors, having achieved higher scores in UMAT-3 tended to mitigate this outcome. The major focus in attempts to grow the rural medical workforce should therefore remain on recruiting medical students from a rural background together with providing maximal opportunity for prolonged immersion in rural clinical environments during their training.

## Background

The state of Western Australia, which occupies a land mass that constitutes approximately a third of the area of Australia and with approximately one tenth of Australia’s population, is encountering increasing difficulties in the provision of adequate health and medical services to what are widely dispersed rural communities. An ongoing major challenge has been to recruit sufficient medical practitioners to service these rural and remote regions. In response to this challenge, and on the basis of mounting evidence that a rural background leads to more students opting for a career in rural practice [1,2], the University of Western Australia (UWA) began a Rural Student Recruitment program [3]. In addition in 2002, on the premise that increased opportunities for student immersion in rural clinical environments would also hopefully enhance the prospects of its graduates choosing to practice rurally after graduation [4], UWA commenced a Rural Clinical School (RCS). In a recent report [5] we have demonstrated that these strategies appear to be achieving the desired effect with students from a rural background 7.5–fold more likely to be practicing in a rural setting than their urban counterparts. Furthermore, students from both urban and rural backgrounds who spend the fifth year of medical school in clinical rotations in our RCS, are approximately 4–fold more likely to be in rural practice 3 or more years after completing their MBBS degrees [5].

A relatively under-explored question, however, is the extent to which other factors may further enhance the chances of medical students choosing to work rurally after graduation. In this respect, we have previously assessed a range of socio-demographic factors together with the selection factors used for medical school entry that might dictate future intentions of newly enrolled medical students to ultimately pursue rural practice [6]. In that study we analysed a cohort of students admitted from 2006 to 2011 who had completed the Medical Students Outcomes Database Questionnaire [7] and answered a question on their intent after graduation to practice in a rural site versus an urban site. The results confirmed the anticipated strong influences of both a rural background effect as well as intention towards generalist rather than specialist practice on intentions to practice rurally. However, the results also suggested that a focus on increasing the recruitment of students from lower socio-economic areas and perhaps setting lower thresholds with respect to the very high Australian Tertiary Admissions Rank (ATAR) scores required for entry, might be further potential strategies to consider in efforts to bolster the rural workforce.

We have now had the opportunity to explore this in a cohort of students who have already graduated from our medical school and who are now in at least their third year in the medical workforce. This has permitted an analysis of the socio-demographic profile of entering medical students together with those factors used in their selection for medical school, as potential predictors of those who ultimately have elected to practice in a rural site.

## Methods

### Study cohort

In 1999 MBBS students entering the University of Western Australia (UWA) Medical School did so via a revised selection process which introduced, in addition to the previous single entry criterion of academic performance as assessed by ATAR [8], a score from a structured interview [9] and the score from an aptitude test - the Undergraduate Medicine and Health Sciences Admission Test (UMAT) [10]. The current study therefore, comprised all standard entrants from secondary school to UWA from 1999 to 2006 who were admitted via the new selection process and who subsequently graduated by 2011, ensuring each graduate was in at least their third post-graduate year at the time of data collection. In February 2014 information was accessed from the Australian Health Practitioner Regulation Agency (AHPRA) database to identify each graduate’s current workplace location. Graduates were designated as working rurally if their primary practice location was in an area defined by the Australian Standard Geographical Classification - Remoteness Area (ASGC-RA) [11] as RA 2–5, and urban if RA 1.

These five geographical areas are based on the Accessibility/Remoteness Index of Australia (ARIA) [11]. ARIA is a continuous varying index with values ranging from 0 (high accessibility) to 15 (high remoteness), and is based on road distance measurements from over 12,000 populated localities to the nearest Service Centres in five size categories based on population size. ASGC-RA 1 refers to the Major Cities of Australia (ARIA score 0–0.2) characterized by relatively unrestricted accessibility to a wide range of goods and services and wide opportunities for social interaction; ASGC-RA 2 refers to Inner Regional Australia (ARIA score >0.2 and ≤2.4) and is characterized by some restrictions to accessibility of some goods, services and opportunities for social interaction; ASGC-RA 3 designates Outer Regional Australia (ARIA score >2.4 and ≤5.92) and is defined by significantly restricted accessibility; ASGC-RA 4 defines Remote Australia (ARIA score >5.92 and ≤ 10.53) with very restricted accessibility and ASGC-RA 5 defines Very Remote Australia (ARIA score >10.53) with very little accessibility.

This study is part of a larger ongoing project which includes the follow-up of all school leaver entrants to the MBBS course at the University of Western Australia. Results of the larger project have been reported elsewhere [9]. The project has been approved by the Human Research Ethics Committee at UWA as an amendment to the larger project (file reference RA/4/1/2178).

### Selection factors

A more detailed description of each of the 3 selection factors (ATAR, standardised interview and UMAT) can be found in our previous reports [9,12,13]. They were weighted in a ratio of 1 : 1 : 1 to determine a final composite selection score from which all applicants were subsequently ranked before selection and final offers of a medical school place. In the current analysis, the three component UMAT scores, UMAT-1 (Logical reasoning and problem solving), UMAT-2 (Understanding people) and UMAT-3 (Non-verbal reasoning), were independently evaluated together with the total score because of their different and independent constructs. The UMAT scale has changed over time and scores are not necessarily comparable between years. Therefore percentile ranks, which enable a measure of the relative standing of a candidate within each annual cohort, have been utilised in this study rather than the raw score.

### Socio-demographic factors

Age, gender and type of school attended - government (publicly funded) or independent (fee paying) - were recorded at entry to medical school. Age at graduation from medical school exhibited marked kurtosis and skew and so has been dichotomised into those 23 yr and younger vs those 24 yr and older. As a socioeconomic indicator, the correspondence postcode at entry for each student was linked to the Index of Relative Socioeconomic Advantage and Disadvantage (IRSAD) score from the Australian 2006 census Socio-Economic Indices for Areas (SEIFA) [14]. The construct for SEIFA codes, and the caveats in relation to their use as socio-economic indicators, have previously been described [12]. A dummy variable was constructed which dichotomised the cohort into the top 4 deciles for IRSAD score vs the bottom 6 deciles. Region of origin was determined from country of origin according to major regional groups as outlined in the Australian Standard Classification of Countries for Social Statistics [15]. Given the relatively small numbers of students in some groups they were collapsed into 5 groups for analysis - those from Oceania (Australia, New Zealand, Papua New Guinea and proximate Pacific islands), UK and Ireland, NE and SE Asia, Southern Asia (India, Pakistan, Sri Lanka and Bangladesh) and Other.

### Rural background and RCS participation

Up until 2007 all applicants to the UWA medical school were considered from a rural background if they had lived in a rural area of Western Australia for a minimum of two years and, during that period, completed year 12 at a rural secondary school – “rural” being defined as a distance of >75 kms from the Perth Central Business District. The utilisation of the ASGC Remoteness Area classification to define rurality did not commence at UWA until 2010. However, all subjects within the above rural definition were from areas designated ASGC-RA 2 to 5 and an ASSGC-RA classification for each student was based on their rural town of origin in the present analysis. Otherwise students were classified as urban background (ASGC-RA 1).

The final cohort was linked to a database of all students who have been enrolled in the RCS since it commenced in 2002 to identify those who completed level 5 of the MBBS course in a rural location. The RCS up to 2011 occupied 13 sites, two designated ASGC-RA 2, three designated ASGC-RA 3, seven designated ASGC-RA 4 and one designated ASGC-RA 5.

### Statistics

Data were analysed using IBM SPSS Statistics Release 20.0.0. All values are reported as Mean ± SEM. Univariate comparisons by rural vs urban background were made using the χ^2^ test for categorical variables and unpaired T-test for each selection factor. Univariate analysis of predictors of practicing in a rural vs urban environment utilised logistic regression for categorical variables and unpaired T-tests for each selection factor. Multivariate logistic regression models were constructed for the major outcome variable of current site of practice in 2014 (urban vs rural), using the full set of selection factors and socio-demographic variables outlined above. Further models included adjustment for the independent and combined influence of both rural background and participation in the RCS on ultimate practice destination.

## Results

### Summary statistics

There were 774 subjects eligible for the analysis of whom 31 could not be traced on the AHPRA database, 10 who were either overseas or registered as non-practicing and 4 who were deferred entrants from 1998 and did not sit the UMAT or interview (Table 1) (final effective sample size N = 729, participation rate 94%). A comparison of the selection scores (UMAT and each of its components, ATAR and interview score) in those who were finally included versus those who did not, revealed no significant differences in ATAR, the interview score, total UMAT percentile score or percentile score in each of its 3 components. There were no significant differences in the distribution for both groups in terms of age, rural background, IRSAD decile and type of high school attended but those not included had a higher proportion of females (χ^2^ = 7.0, P = 0.005).

**Table 1:** **Description of University of Western Australia standard pathway entrants from 1999 who graduated by 2011**

	Year of completion of course								
	2004	2005	2006	2007	2008	2009	2010	2011	Total
Graduates	60	81	89	93	106	123	125	97	774
Ineligible for inclusion									
Overseas or Non-practicing	2	4	3	0	0	1	0	0	10
Deferred entry – ATAR alone	2	2	0	0	0	0	0	0	4
Unable to be traced	4	6	1	7	7	3	2	2	31
Included in the study	54	71	85	86	99	120	123	95	729

The final cohort had a mean age of 23.6 ± 0.04 yr at completion of the course. Approximately 86% were of urban origin (N = 630) and 14% of rural origin (N = 99), 55% were female (N = 404) and 45% were male (N = 325), 63% were from an independent school background (N = 420) and 37% from a Government school background (N = 243), 12.5% were from the lower 6 IRSAD deciles (N = 91) and 87.5% from the upper 4 deciles (N = 638). This profile was not significantly different for each year of the cohort from 1999 to 2006.

Selection criteria and socio-demographic factors by rural vs urban background are listed in Table 2. Rural students had lower academic entry scores and UMAT percentile scores but interview scores were not significantly different. Rural students were older and more likely to come from an area of reduced socio-economic advantage and increased socioeconomic disadvantage. Rural students included more females and were more likely to have attended Government rather than independent schools and were twice as likely to have been enrolled in the RCS during the 5^th^ year of the course.

**Table 2:** **Selection criteria and socio-demographic factors by rural vs urban background**

Variable	N	Urban background	N	Rural background	P-value (χ ^ 2 ^ test)
**Academic entry score (ATAR)**	630	99.0 ± 0.04	99	97.50 ± 0.18	**<0.001**
**Interview score**	630	28.0 ± 0.2	99	26.9 ± 0.6	0.065
**Total UMAT Percentile score**	630	84.6 ± 0.4	99	75.8 ± 2.0	**0.022**
**UMAT-1 Percentile score**	629	79.7 ± 0.7	99	74.5 ± 2.1	**0.865**
**UMAT-2 Percentile score**	629	72.8 ± 0.9	99	73.2 ± 2.2	**<0.001**
**UMAT-3 Percentile score**	629	77.5 ± 0.8	99	63.4 ± 2.5	**0.009**
**Age at completion**					**0.022**
Up to 23 yr	377	59.8%	48	48.5%	
24 yr and older	253	40.2%	51	51.5%	
**Sex**					**0.010**
Female	338	53.7%	66	66.7%	
Male	292	46.3%	33	33.3%	
**Secondary school**					**<0.001**
Government	189	33.4%	54	55.7%	
Independent	377	66.6%	43	44.3%	
**IRSAD score**					**<0.001**
Deciles 1-2	4	0.6%	3	3.0%	
Deciles 3-4	7	1.1%	12	12.1%	
Deciles 5-6	29	4.6%	36	36.4%	
Deciles 7-8	93	14.8%	19	19.2%	
Deciles 9-10	497	78.9%	29	29.3%	
**ASGC-Remoteness area**					**<0.001**
Major Cities	621	98.6%	0	0	
Inner regional	4	0.6%	35	35.4%	
Outer regional	4	0.6%	51	51.5%	
Remote	1	0.2%	11	11.1%	
Very remote	0	0	2	2.0%	
**Country of origin**					**<0.001**
Oceania	397	63.0%	88	88.9%	
UK and Ireland	25	6.3%	1	1.0%	
Eastern and SE Asia	116	18.4%	4	4.0%	
Southern Asia	40	6.3%	1	1.0%	
Other	52	8.3%	5	5.1%	

Significant P values are in bold-faced type.

### Univariate analysis of the selection factors

Because rural students were recruited with lower overall academic entry scores and UMAT scores, univariate analyses of the selection factors for each group were conducted separately (Tables 3 and 4). In urban background students, the academic entry score and interview score were no different between those currently working in a rural area vs those in an urban site of practice. The percentile scores for UMAT-1 and UMAT-2 were also similar, while for UMAT-3, scores were lower in those currently in a rural site (Table 3). For rural background students, academic entry score, interview and total UMAT percentile score as well as scores in each of its components were no different in those currently working rurally vs those in an urban site of practice (Table 4).

**Table 3:** **Selection factors by current site of practice in urban background subjects**

	Current site of practice	N	Mean ± SEM	P value (Unpaired T-Test)
Academic entry score (ATAR)	Urban	587	98.98 ± 0.04	0.210
	Rural	43	98.80 ± 0.16	
Interview score	Urban	587	28.0 ± 0.2	0.731
	Rural	43	28.3 ± 0.7	
Total UMAT Percentile score	Urban	587	84.8 ± 0.5	0.068
	Rural	43	81.6 ± 1.7	
UMAT-1 Percentile score	Urban	586	77.4 ± 2.7	0.366
	Rural	43	79.9 ± 0.7	
UMAT-2 Percentile score	Urban	586	72.8 ± 0.9	0.757
	Rural	43	71.8 ± 3.0	
UMAT-3 Percentile score	Urban	586	78.1 ± 0.8	**0.046**
	Rural	43	69.6 ± 4.1	

Significant P values are in bold-faced type.

**Table 4:** **Selection factors by current site of practice in rural background subjects**

	Current site of practice	N	Mean ± SEM	P value (Unpaired T-Test)
Academic entry score (ATAR)	Urban	78	97.54 ± 0.20	0.677
	Rural	21	97.36 ± 0.45	
Interview score	Urban	78	26.9 ± 0.7	0.731
	Rural	21	27.0 ± 1.1	
Total UMAT Percentile score	Urban	78	75.4 ± 2.2	0.677
	Rural	21	77.4 ± 4.7	
UMAT-1 Percentile score	Urban	78	74.7 ± 2.4	0.827
	Rural	21	73.6 ± 4.9	
UMAT-2 Percentile score	Urban	78	72.5 ± 2.5	0.503
	Rural	21	76.0 ± 4.4	
UMAT-3 Percentile score	Urban	78	63.3 ± 2.9	0.955
	Rural	21	63.7 ± 4.6	

### Univariate analysis of the socio-demographic data

Univariate analysis of socio-demographic variables in the prediction of the likelihood of students working in a rural site vs an urban environment are outlined in Table 5. Together, being from rural background and having attended the RCS predicted a nearly 10-fold increase in the odds of currently practicing rurally compared to those who were from an urban background and hadn’t attended the RCS. Being from an urban background but having attended the RCS predicted a 3.31-fold increase in the odds of currently practicing rurally, while being from an rural background but not having attended the RCS predicted a 2.83-fold increase in the odds of currently practicing rurally. For rural background students alone, increasing rurality, as measured by ASGC-RA 1–5 for the town of origin at entry to medical school, did not further increase the likelihood of practicing rurally (Table 5). There was however, a significant and increasing trend for those from the lower 6 IRSAD deciles to be currently working rurally compared to those in the upper 4 deciles. Students from Government versus independent schools had a small increase in the odds of current rural practice which was of borderline significance only.

**Table 5:** **Univariate predictors of graduates currently working in a rural environment**

	Number (%) currently in rural site of practice	Odds ratio (Logistic regression)	*P*
**Rural background/RCS**			
Urban and RCS non-participant	21/467 (4.5%)	1.0	
Rural and RCS non-participant	6/51 (11.8%)	2.83 (1.09, 7.38)	**0.033**
Urban and RCS participant	22/163 (13.5%)	3.31 (1.77, 6.21)	**<0.001**
Rural and RCS participant	15/48 (31.2%)	9.65 (4.56, 20.46)	**<0.001**
**Age at completion**			
Up to 23 yr	27/425 (6.4%)	1.0	
24 yr and older	37/304 (12.2%)	2.04 (1.22, 3.44)	**0.007**
**Sex**			
Female	37/404 (9.2%)	1.11 (0.66, 1.87)	0.687
Male	27/325 (8.3%)	1.0	
**IRSAD score**			
Deciles 1-2	2/7 (28.6%)	5.44 (1.02, 29.05)	**0.047**
Deciles 3-4	4/19 (21.1%)	3.63 (1.15, 11.51)	**0.029**
Deciles 5-6	12/65 (18.5%)	3.08 (1.51, 6.28)	**0.002**
Deciles 7-8	10/112 (8.9%)	1.33 (0.64, 2.78)	0.440
Deciles 9-10	36/526 (6.8%)	1.0	
**ASGC-Remoteness area**			
Major Cities	42/621 (6.8%)	1.0	
Inner regional	9/39 (23.1%)	4.14 (1.84, 9.28)	**<0.001**
Outer regional	12/55 (21.8%)	3.85 (1.89, 7.84)	**<0.001**
Remote and Very remote	1/14 (7.1%)	1.06 (0.14, 8.30)	0.955
**Secondary school**			
Government	27/243 (11.1%)	1.69 (0.97, 2.92)	0.056
Independent	29/420 (6.9%)	1.0	
**Country of origin**			
Oceania	52/485 (10.7%)	1.0	
UK and Ireland	2/26 (7.7%)	3.30 (0.78, 13.94)	0.104
Eastern and SE Asia	6/120 (5.0%)	2.29 (3.10, 17.24)	0.421
Southern Asia	2/41 (4.9%)	1.45 (0.28, 7.40)	0.657
Other	2/57 (3.1%)	1.41 (0.19, 10.45)	0.737

Significant P values are in bold-faced type.

### Multivariate analyses of selection factors and socio-demographic data

The final multivariate logistic regression model is outlined in Table 6. Those practicing in a rural location in 2014 were more likely to come from the lower 6 IRSAD deciles on admission to medical school (OR 2.75, 95% CI 1.44, 5.23, P = 0.002), to be older (OR 1.86, 95% CI 1.09, 3.18, p = 0.023) and to have a lower UMAT-3 (Non-verbal communication) percentile score (OR 0.98, 95% CI 0.97, 0.99, P = 0.005). In a separate analysis (Table 7), both rural background and participation in the Rural Clinical School were first forced into the model to control for these established influences on the actual likelihood of working rurally after graduation. Being from an urban background but attending the RCS was associated with a more than 3-fold increase in the odds of practicing rurally (P < 0.001) while having a rural background and attending the RCS was associated with a more than 5-fold increase in the odds. After controlling for these influences, only age (P = 0.035) and UMAT-3 percentile score (P = 0.027) remained as independent predictors of rural practice. The further entry of country of origin into the model made no substantial change in these parameter estimates (data not shown).

**Table 6:** **Multivariate logistic regression with current urban vs rural site of practice as the dependent variable and selection factors and socio-demographic variables as the predictor variables (N = 728) (Nagelkerke R Square = 0.09)**

Predictor variable	B	S.E.	P value	Odds ratio	95% CI for odds ratio	
					Lower	Upper
Age at completion						
23 yr or younger				1		
24 yr or older	0.622	0.274	**0.023**	1.86	1.09	3.18
Sex						
Male				1		
Female	−0.119	0.287	0.679	0.89	0.51	1.56
IRSAD score						
Deciles 7-10				1		
Deciles 1-6	1.011	0.329	**0.002**	2.75	1.44	5.23
ATAR	−0.106	0.104	0.308	0.90	0.73	1.10
Interview score	−0.003	0.024	0.888	1.00	0.95	1.05
UMAT-1 Percentile score	−0.009	0.007	0.225	0.99	0.98	1.01
UMAT-2 Percentile score	−0.004	0.006	0.536	1.00	0.98	1.01
UMAT-3 Percentile score	−0.016	0.006	**0.005**	0.98	0.97	1.00

Significant P values are in bold-face type.

**Table 7:** **Multivariate logistic regression with current urban vs rural site of practice as the dependent variable and rural background and RCS participation, selection factors and socio-demographic variables as the predictor variables (N = 728) (Nagelkerke R Square = 0.149)**

Predictor variable	B	S.E.	P value	Odds ratio	95% CI for OR	
					Lower	Upper
Rural background and RCS participation						
Urban and no RCS participation				1		
Rural and no RCS participation	0.388	0.571	0.497	1.48	0.48	4.52
Urban and RCS participation	1.213	0.330	**<0.001**	3.36	1.76	6.42
Rural and RCS participation	1.631	0.487	**0.001**	5.11	1.97	13.25
Age at completion						
23 yr or younger				1		
24 yr or older	0.586	0.278	**0.035**	1.80	1.04	3.10
Sex						
Male				1		
Female	−0.322	0.297	0.278	0.73	0.41	1.30
IRSAD Score						
Deciles 7-10				1		
Deciles 1-6	0.562	0.390	0.150	1.75	0.82	3.78
ATAR	−0.093	0.117	0.429	0.91	0.72	1.15
Interview score	−0.011	0.025	0.662	0.99	0.94	1.04
UMAT-1 Percentile score	−0.008	0.008	0.261	0.99	0.98	1.01
UMAT-2 Percentile score	−0.001	0.007	0.856	1.00	0.99	1.01
UMAT-3 Percentile score	−0.013	0.006	**0.027**	0.99	0.98	1.00

Significant P values are in bold-face type.

## Discussion

It has previously been noted that selection processes into medicine have been relatively neglected as a line of investigation for increasing our understanding of potential predictors of taking up a rural career [16]. We have now assessed whether there is any predictive value in the 3 selection factors that have been utilised at our medical school since 1999, ascertaining the degree to which they might predict current site of practice in all students who were now within at least 3 years of their graduation. After controlling for the multiplicative effects of a prolonged rural undergraduate experience during clinical training and previous rural background, no predictive value was seen for either prior academic performance at secondary school or for score on a standardised interview. Similarly no predictive value was seen for the total score on an aptitude test – the UMAT – or 2 of its components, UMAT-1 (Logical reasoning and problem solving) and UMAT-2 (Understanding people). In contrast, UMAT-3 (Non-verbal reasoning) remained an independent, albeit weak, predictor of current rural practice with an estimate from the regression modelling that a 10 percentile increment in the UMAT-3 score would predict a 13% reduction in the odds of a rurally-based career. We have previously reported that a weaker performance in UMAT-3 is seen in females compared to males, in older subjects and in those with a rural background but a stronger performance by those from Asian language backgrounds [12]. In the current study, the negative predictive effect of a higher UMAT-3 score was still present after adjustment for age and gender and region of origin. It was only significant in urban, not rural background students, however, with modelling in these subjects indicating a 20% reduction in the odds of being in rural practice with each 10 percentile increment in the UMAT-3 score. UMAT-3 is the one component of this aptitude test that appears most susceptible to enhanced performance after prior coaching [17-19]. Given the costs of such coaching it is conceivable that students from areas of social disadvantage or government vs independent schools might have had less access to such coaching, offering a possible explanation, at least in part, for our observation. However, lower UMAT-3 percentile scores were still predictive of rural practice even after these considerations were taken into account.

No difference was seen in academic entry scores between those who were now in a rural practice setting vs an urban location in either rural or urban background students. This result is discordant from that in our previous report [6] which ascertained the predictive value of selection factors and a number of socio-demographic variables on the intention of entering medical students to practice rurally. In that study lower academic entry scores (especially among urban students) were independently associated with a higher likelihood of an intention to practice rurally. In the current study, all subjects entering from secondary school from 1999 were eligible for inclusion if they were now at least in their 3^rd^ year post graduation, while the eligible subjects for our previous report were all those who entered from secondary school from 2006 to 2011. A comparison of the 2 cohorts reveals that although gender mix and socioeconomic background in the 2 cohorts were similar, participants in the current study were younger, less likely to have come from a rural background and more likely to have come from a government school background, all factors which may have impacted on academic entry scores as relative predictors of intended or actual rural practice.

Very few other studies have evaluated the extent to which commonly utilised approaches for student selection may also impact on the ultimate intended or actual destination of practice. In a previous study from our medical school, in an era when selection was based on academic merit alone, in 2 cohorts of students who commenced medical school in 1984 and 1989 respectively, admission scores were not found to be predictive of either rural practice within 4 years of graduation or a generalist vs specialist career choice [20]. Pearson et al. [21], in another Australian study, compared graduate destinations of students from a medical school that used traditional academic criteria alone for student selection, to one that utilised an alternative ‘composite entry’ pathway that also included a psychometric test and an interview. The results were suggestive that choosing students through the ‘composite entry’ pathway resulted in more who chose family medicine and psychiatry as career destinations, but there was no evidence of an influence on ultimate rural versus urban practice location. Rabinowitz and colleagues [22] followed up 3414 graduates from the Jefferson Medical College in Philadelphia and looked at 19 variables that might potentially influence likelihood of becoming a rural primary care physician. These included the scores from an aptitude test - the Medical College Admission Test, as well as the undergraduate science grade point average but neither of these selection factors for these graduate entry students were shown to have any significant predictive value.

We have previously reported that for entering medical students at UWA, an intent to practice rurally was independently predicted by IRSAD - a composite score which includes indicators of both higher socio-economic disadvantage and reduced socio-economic advantage [6]. In the current study, although IRSAD demonstrated a similar univariate trend for actual site of practice after graduation rather than just intent, this was no longer an independent predictor after rural background and participation in our rural clinical school were entered into multivariate models. This reflects the observation that rural students in this cohort were more likely to have come from areas of reduced socio-economic advantage and increased socioeconomic disadvantage. It may also reflect the fact that responses to a question on intended future site of practice at entry to medical school may have been dictated at least in part by idealistic motivations. A decline in idealism as a student progresses through a medical course has been well documented [23,24] and may have been another factor to consider in explaining differences in our 2 study outcomes, with initial intentions to practice in under-served and disadvantaged rural communities subsumed by motivations that become increasingly influenced by social prestige in relation to career choice and specialisation, increasing student debt and desire for a higher income or better job security. In the previously discussed Jefferson Medical College study [22], the only socio-economic variables evaluated were maternal and paternal education, anticipated income bracket on graduation and anticipated proportion of low income patients in their future practice and none of these independently predicted ultimately practicing in primary rural care.

In the present study there was no gender difference in terms of those in rural practice after graduation. In 2008, in the Medicine in Australia: Balancing Employment and Life (MABEL) study, a prospective cohort of over 10,000 Australian doctors was studied in relation to type and location of practice [25] and males were found to have nearly twice the odds of females to be practicing in rural areas. Other studies have also indicated that men rather than women are more likely to enter rural practice [26] although a previous systematic review of determinants of choosing to practice rurally, indicated that this has not been a consistent finding [2]. Following changes to our selection processes in 1999 [13], the increase through subsequent cohorts of medical students in the proportion of females (including numbers of females from a rural background), is likely to have been a factor in our neutral result.

### Limitations of the study

The relatively small overall numbers, the different sizes of each annual intake as well as slight variation each year in UMAT scores, interview scores and ATAR for each cohort are limitations in this study. The use of the AHPRA data-base to assess current practice location may have failed to capture shorter periods of rural practice either before or during 2014 and probably under-estimates ultimate rural practice destination. The use of an individual’s postcode as a surrogate for socio-economic status imputes an index based on the level of socio-economic disadvantage for all people living in a defined area and may not be truly reflective of socio-economic status for each individual [14].

## Conclusion

Once the influence of a prolonged immersion in a rural clinical environment during undergraduate clinical training as well as the effect of coming from a rural background were considered, we were able to demonstrate very little in the way of a further influence of medical school selection factors or student socio-demographic profiles on the likelihood of ultimately taking up rural practice after graduation. Among the 3 selection factors utilised for entry into our medical school from secondary school (ATAR, interview and UMAT), only a lower percentile score in UMAT-3 weakly predicted a current rural vs urban site of practice. In terms of the socio-demographic profiles of those selected, only older age weakly augmented the selection of graduates likely to ultimately work in a rural destination. These results indicate that the major focus of medical school initiatives to grow the rural medical workforce should remain on recruiting medical students from a rural background together with providing maximal opportunity for prolonged clinical clerkships in rural environments for as many students as possible during their training.
